# Public perception towards marriage involving individuals with schizophrenia and its relationship with affirming attitudes among the general population

**DOI:** 10.1192/j.eurpsy.2025.2275

**Published:** 2025-08-26

**Authors:** Y. Trabelsi, B. N. Saguem, N. Jaafar

**Affiliations:** 1 Psychiatry Department, Farhat Hached Hospital, sousse, Tunisia

## Abstract

**Introduction:**

The perception of marriage involving individuals with schizophrenia remains critical in understanding the broader societal attitudes towards mental illness. Stigma and misconceptions often shape public views. Few studies have been conducted on this subject and the perceptions regarding this topic have not been thoroughly investigated.

**Objectives:**

This study aimed to explore perceptions of the general public regarding marriage in individuals with schizophrenia and to assess the relationships between these perceptions and affirming attitudes.

**Methods:**

A cross sectional study was conducted via an online survey. It included a detailed description of clinical symptoms and outcomes of schizophrenia. Thirteen open-ended questions, adopted from the study of Kumar et al., 2019, and assessing various aspects of marriage in individuals with schizophrenia were asked.

A battery for measurement of affirming attitudes about mental illness was used, comprising the Empowerment scale(ES) to assess people’s beliefs about the social worth of people with mental illness, the Recovery scale (RS) to evaluate people’s beliefs about potential of recovery from serious mental illness and the self-discrimination scale (SDS) to assess people’s expectations about a person with mental illness successfully pursuing his life goals.

**Results:**

A total of 304 participants took part in the study, most of whom were between 20 and 30 years old, 80,9 were women. Additionally, 23.35% mentioned living with someone diagnosed with a psychiatric disorder. Results revealed that the majority of participants held reservations about marriage for individuals with schizophrenia. Over 50% believed that marriage neither cures nor improves symptoms, and more than 60% were reluctant to marry someone with schizophrenia or agreed to a marriage match involving a schizophrenic person. However, 53% agreed that individuals with schizophrenia have the right to make their own decisions about marriage, though concerns remain regarding their ability to manage family responsibilities. Regarding perceptions of schizophrenia, individuals who believed that marriage is not a cure for the illness and those who stated they would never consider marrying someone with schizophrenia exhibited significantly higher RS scores (p=.009 and p<10^-3, respectively). Participants who disagreed with the fact that individuals with schizophrenia have the right to marry showed significantly higher RS (p=.023), ES (p=.03), and SDS (p=.033) scores.

**Conclusions:**

This study highlights the ongoing challenge of stigma towards individuals with schizophrenia, particularly about their perceived suitability for marriage. While there are encouraging signs of changing attitudes, broader efforts are needed to foster a more inclusive and supportive societal perspective on mental health and marriage.

**Disclosure of Interest:**

None Declared

